# Effect of combination therapy with ceftizoxime and clotrimazole in the treatment of otomycosis 

**DOI:** 10.18502/cmm.4.1.30

**Published:** 2018-03

**Authors:** Saeid Mahdavi Omran, Zahra Yousefzade, Soraya Khafri, Mojtaba Taghizadeh-Armaki, Keyvan Kiakojuri

**Affiliations:** 1 Infectious Diseases and Tropical Medicine Research Center, Health Research Center, Department of Parasitology and Mycology, School of Medicine, Babol University of Medical Sciences, Babol, Iran; 2 Department of Medical Parasitology and Mycology, Faculty of Medicine, Babol University of Medical Sciences, Babol, Iran; 3 Department of Biostatistics, Faculty of Medicine, Babol University of Medical Sciences, Babol, Iran; 4 Department of Ear, Nose, and Throat, Faculty of Medicine, Roohani Hospital, Babol University of Medical Sciences, Babol, Iran

**Keywords:** Ceftizoxime, Clotrimazole, Middle ear, Otomycosis, Tympanic membrane rupture

## Abstract

**Background and Purpose:**

There are controversial findings regarding the efficacy of antifungal drugs in the treatment of a ruptured eardrum following fungal infections. Regarding this, the aim of the present study was to evaluate the therapeutic effect of the co-administration of antifungal and antibacterial agents in the treatment of otomycosis with tympanic membrane perforation.

**Materials and Methods:**

This analytical, clinical trial was conducted on 87 patients with otomycosis showing no bacterial elements in the direct observation and culture. The study population was assigned into two groups of intervention (n=45) and control (n=42). The demographic and clinical data, as well as the data related to the direct observation and culture of the ear samples were recorded in a checklist. All statistical analysis was performed in SPSS (version 24).

**Results:**

The most prevalent symptoms in both groups were hearing loss and itching, and the most common finding was secretion. *Aspergillus* and *Candida* were the most frequent fungi isolated from the samples. After the implementation of combination therapy, the intervention group demonstrated a significant decrease in symptoms and signs, compared to the control group (*P=0.005*).

**Conclusion:**

The findings of the present study indicated that the use combination therapy with ceftizoxime powder and clotrimazole ointment was effective the in treatment of the patients with tympanic membrane rupture showing no bacterial effects in direct examination and culture.

## Introduction

Otomycosis is often referred to the superficial fungal infection of the auricle and external auditory canal, though this disease can also affect the middle ear in case of tympanic membrane perforation [1-4]. Otomycosis is rarely life-threatening; however, the course of this disease is challenging and exhausting due its long-term treatment and follow-up, as well as the high probability of recurrence [5].

There is a controversy over the incidence of otomycosis that whether it is caused by a fungal agent or occurs after a secondary bacterial infection. Nonetheless, the majority of the clinical and laboratory evidence has shown otomycosis as a true pathologic entity [6, 7]. The most common etiologic fungal agents causing otomycosis are *Aspergillus* and *Candida* species [7-9]. 

The onset of clinical symptoms is usually associated with pruritus, hearing loss, erythema, and outer ear inflammation [8-10]. The predisposing factors for otomycosis include climate change, moisture, presence of cerumen, ear manipulation, weakened immune system, and long-term use of broad-spectrum antibiotics and steroids [7, 10].

A diversity of topical antifungal agents has traditionally been used alone or in combination to treat otomycosis [11]. The main management of ear fungal infection is the elimination of noticeable debris and fungal elements [12]. The topical therapeutic agents suggested for the control of this condition include steroids, acidic solutions, antiseptics, and antifungal drugs [13]. The antifungal agents commonly applied for ear fungal infection do not always overcome the disease. The treatment of this infection should also improve the physiological symptoms of the external ear canal [12]. 

The administration of boric acid in an alcohol solution for the treatment of otomycosis is reported to result in a recurrence rate of 23% [12, 14]. In addition, the use of antifungal drug solutions, such as nystatin and clotrimazole, may be effective for the management of otomycosis [14, 15]. There are several reports about the resistance of *Aspergillus* and *Candida* species isolated from otomycosis to current therapies [12, 16, 17]. 

Consequently, it is essential to find a suitable therapeutic regimen for this infection [5]. Recently, the use of combined antifungal and antibiotic therapy has been reported as a successful treatment of otomycosis [18]. With this background in mind, the present study aimed to evaluate the therapeutic effect of the co-administration of antifungal and antibacterial agents in the treatment of otomycosis with tympanic membrane perforation.

## Materials and Methods

The current analytical, clinical trial was performed in the Ear, Nose, and Throat Clinic of Ayatollah Rouhani Hospital of Babol, Iran, in 2015-2017. This study was approved by the Ethics Committee of Babol University of Medical Sciences (mubabol. rec. 1395.28). The present clinical trial was registered in the Iranian Registry of Clinical Trials (www.irct.ir; Trial no: IRCT2016082313136N4; registration date: October 13, 2016).

A total of 108 ear samples were collected from 106 patients with otomycosis associated with tympanic membrane perforation. The exclusion criteria were: 1) narrow external auditory canals, 2) tympanic membrane perforation caused by trauma, 3) active middle ear infection, 4) weakened immune system (e.g., uncontrolled diabetes and bacterial infection), 5) absence of fungi in the direct examination, and 6) unwillingness to continue participating in the study.

The clinical symptoms and signs were evaluated by an ENT specialist in two visits. These data were documented by a questionnaire. The pus/discharge samples were taken aseptically from the middle ear of the patients using a speculum, suction, curette, or loop. The patients were randomly divided into two groups of control and intervention. After performing suction clearance, both groups were injected with clotrimazole 1% cream (Pars Darou Pharmaceutical Company) into the middle ear canal using an 18G PVC needle under the microscopic guide. 

Subsequently, the intervention group received ceftizoxime 1 g powder (Jaber Ebne Hayyan Pharmaceutical Company, Iran) sprayed in the ear canal using insufflation. The cases showing the elimination of clinical symptoms, such as pus and discharge, from ear infection were considered as the ones responding to treatment. The samples were taken from the ear canal and prepared for direct examination and culture in the mycology laboratory of Babol University of Medical Sciences. The slides were stained with methylene blue dye. 

The presence of true or pseudo mycelia, yeast cells, as well as reproductive organs indicated that the specimens were positive for fungi at this stage. A portion of samples were cultured on specific bacteria media, such as chocolate agar and blood agar. The incubation time was 48 h at 37°C. The colonies were identified according to biochemical and microbial criteria. The remaining samples were cultured on Sabouraud dextrose agar supplemented with chloramphenicol. The plates were incubated at room temperature for at least 4 weeks. The fungal colonies were identified by macroscopic and microscopic criteria.

***Statistical analysis***

The data related to patients and laboratory results were analyzed in SPSS software (version 24) using independent t-test, Chi-Square test, and McNemar's test. The comparison of the difference in the symptoms and signs results between the first and second visits was accomplished by means of the Mann-Whitney U test, which revealed the effect of the drug on patients' condition in the two groups. *P-value* less than 0.05 was considered statistically significant.

## Results

Out of 108 samples, 21 cases were excluded due to the presence of bacteria or showing negative results for fungal element in direct examination and culture. Finally, 45 and 42 ear samples were collected from the intervention and control groups, respectively. The mean ages of the intervention and control groups were 50.2 and 49.5 years, respectively. Furthermore, 75% (n=36) and 62.5% (n=25) of the intervention and control groups were female, respectively. 

According to Table 1, there was no significant difference between the two groups in terms of the demographic data (*P>0.05*). Our results indicated that the number of the women were higher than that of the men.

In the present study, otomycosis had the female to male ratio of 2:1. In both groups, housekeeping was the most frequent occupation (50.6%), and 69.6% of all patients were living in urban areas. 

About 63.6% of the patients in both groups had ear manipulation, and most of the manipulations were related to the use of cotton swabs (Table 2).** Our results indicated that **swelling had the lowest percentage in both groups (*P=0.96*) (Table 3). In most of the cases, the left ear was the involved ear with the frequencies of 55.6% and 61.1% in the intervention and control groups, respectively (*P>0.05*). 

**Figure 1:**
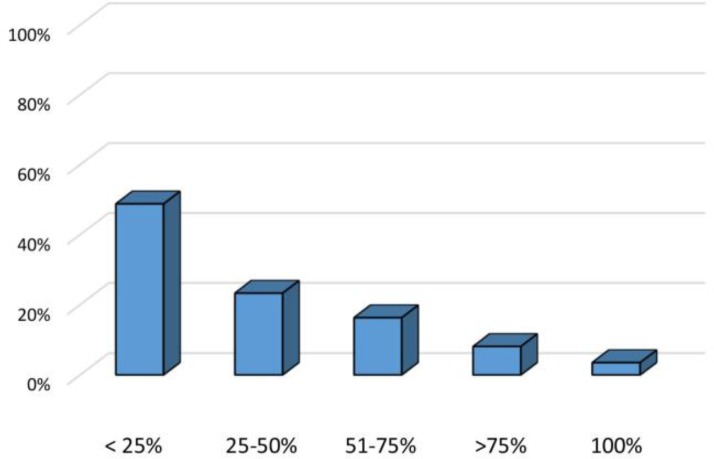
Size of tympanic membrane perforation in the studied groups

**Figure 2:**
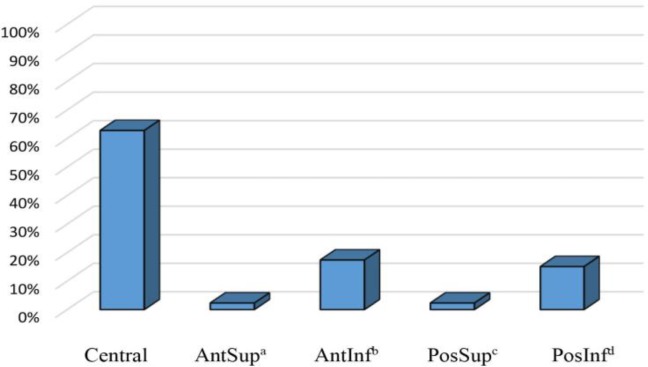
Prevalence of the site of tympanic membrane perforation in the studied groups

Regarding the size of tympanic membrane perforation, it was < 25% in the majority of the patients in both groups. In this regard, only three patients showed complete tympanic membrane perforation (Figure 1). The most common site of tympanic membrane perforation was the central region, accounting for 62.8% of the patients. On the other hand, the most uncommon sites of rupture were the anterior and posterior upper regions (Figure 2). 

**Table 1:** Demographic data of the intervention and control groups

**Variable**	**Sub-group**	**Case**	**Control**	***P-value***	**Total**
Gender	Male	12 (26.7%)	15 (37.5%)	*P>0.05*	27 (38.1%)
	Female	33 (73.3%)	25 (62.5%)		58 (68.2%)
Occupation	Unemployed	3 (6.7%)	5 (12.5%)	*P>0.05*	8 (9.4%)
	Student	1 (2.2%)	2 (0.5%)		3 (3.5%)
	Housewife	23 (51.1%)	20 (50%)		43 (50.6%)
	Employee	3 (6.7%)	5 (12.5%)		8 (9.4%)
	Free job	13 (28.9%)	8 (20%)		21 (24.7%)
	Farmer	2 (4.4%)	0 (0%)		2 (2.4%)
Place of residence	City	29 (64.44%)	29 (72.5%)	*P>0.05*	58 (68.24%)
	Village	16 (35.56%)	11 (27.5%)		27 (31.76%)
Ear involved	Right	20 (44.4%)	16 (38.1%)*	*P>0.05*	36 (41.4%)
	Left	25 (55.6%)	26 (61.1%) *		51 (58.6%)
Total	-	45 (51.7%)	42 (48.3%) *	-	87 (100%)

* There are 40 patients in the control group; in two cases, two samples were taken from both ears, so the total number of samples was 42.

**Table 2:** Types of ear manipulation in the studied groups

**Criteria**	**Sub-group**	**Control**	**Case**	**Total**
Type of manipulation	Cotton swab	18 (72.0 %)	16 (66.7 %)	34 (69.3 %)
	Matchwood	2 (8.0 %)	3 (12.5 %)	5 (10.2 %)
	Both cotton swab and matchwood	5 (20.0 %)	4 (16.7 %)	9 (18.3 %)
	Others	0 (0.0 %)	1 (4.2 %)	1 (2.0 %)

**Table 3:** Comparison of clinical symptoms in the two groups between the first and second visits

**Variable**	**Group**								
	**Sub-groupgroup**	**Case**				**Control**			
		**First visit**	**Second Visit**	***P-value***	**Differences between the first and second visits**	**First visit**	**Second visit**	***P-value***	**Differences between the first and second visits**
Pain	Yes	35 (77.8 %)	5 (11.1%)	> 0.001	-0.66±0.47 (-1)*	27 (64.3%)	16 (38.1%)	> 0.001	-0.26±0.44 (0) *
	No	10 (22.2 %)	40 (88.9%)			15 (35.7%)	26 (61.9%)		
Swelling	Yes	26 (57.8 %)	1 (2.2%) *	> 0.001	-0.55±0.51 (-1) *	29 (69.0%)	22 (52.4%)	0.016	0.16±0.37 (-1) *
	No	19 (42.2 %)	44 (97.8%)			13 (31.0%)	20 (47.6%)		
Itching	Yes	38 (84.4 %)	7 (15.6%) *	> 0.001	-0.68±0.46 (-1) *	38 (90.5%)	35 (83.3%)	0.25	-0.07±0.26 (0)*
	No	7 (15.6 %)	38 (84.4%)			4 (9.5%)	7 (16.7%)		
Secretion	Yes	39 (86.7%)	0 (0.0%) *	-	-0.86±0.34 (-1) *	36 (87%)	35 (83.3%)	0.5	-0.04±0.21 (0) *
	No	7 (13.3 %)	45 (100%)			5 (12.2%)	7 (16.7%)		
Hearing loss	Yes	43 (95.6%)	23 (51.1 %) *	> 0.001	-0.44±0.5 (0) *	40 (95.2%)	40 (95.2%	1.0	-0.0±0.0 (0)*-0.0±0.0 (0) *
	No	2 (4.4%)	22 (48.9%)			2 (4.8%)	2 (4.8%)		

* There was a significant difference between the symptoms of the patients in the case and control groups at the level of α=0.05

The results indicated a significant decrease in the clinical symptoms of the intervention group after the administration of clotrimazole cream and ceftizoxime powder (Table 3). However, the control group showed a significant decrease in the pain and swelling (Table 4). *Aspergillus flavus,* *A. niger*, *Candida* species, *A. fumigatus*, and the rest of the fungus had the frequencies of 33.3%, 29.9%, 21.8%, 8%, and 7%, respectively. 

A; Anterior Superior, B; Anterior Inferior, C; Posterior Superior, D; Posterior Inferior.

**Table 4:** Comparison of the signs in the two groups between the first and second visits

**Variable**	**Sub-group**	**Case**				**Control**			
		**First visit**	**Second Visit**	***P-value***	**Differences between the first and second visits**	**First visit**	**Second Visit**	***P-value***	**Differences between the first and second visits**
Swelling and inflammation	Yes	33 (73.3%)	4 (8.9%)	< 0.001	-0.64±0.48 (-1)*	32 (76.2%)	14 (33.3%)	< 0.001	-0.42±0.5 (0)
	No	12 (26.7%)	41 (91.1%)			10 (23.8%)	28 (66.7%)		
Secretion	Yes	33 (77.3%)	3 (6.7%)	< 0.001	-0.66±0.47 (-1)*	41 (97.6%)	35 (83.3%)	< 0.031	0.14±0.33 (0)
	No	12 (26.7%)	42 (93.3%)			1 (2.4%)	7 (16.7%)		

* There was a significant difference between the symptoms of the patients in the case and control groups at the level of α=0.05

## Discussion

Otomycosis is a fungal infection with a worldwide distribution [17]. The US food and drug administration (FDA) has approved several ear antifungal agents for the management of otomycosis [5]. The azole antifungals, such as clotrimazole, miconazole, ketoconazole, and fluconazole, are more useful for the treatment of ear fungal infection and have no ototoxicity [19]. The aim of the present study was to determine the role of the combined use of two drugs in the treatment of the patients with otomycosis. 

As the findings of the present study indicated, the patients treated with ceftizoxime powder, along with clotrimazole ointment, had significantly lower symptoms than those managed with clotrimazole ointment alone. In line with our findings, in a single-blind randomized clinical study, 93.3% of the cases treated with clotrimazole and lignocaine solutions demonstrated an improvement in the clinical symptoms of itching and discharge, and ear pain was recovered in 86.7% of the patients [20]. In another study, Aju and Sagesh (2017) used a combination therapy with oral itraconazole and ofloxacin ear drops for the treatment of otomycosis [21] and reported similar results. 

On the other hand, Kiakojouri et al. (2015) observed no improvement regarding the ear fungal infection in neither intervention nor control groups after two weeks of using boericke alcohol, along with miconazole ointment [22]. In the present study, the most common symptoms were hearing loss, pruritus, and discharge. In several studies conducted in Thailand (2016), Pakistan (2014), and China (2012), the aural fullness (91.2%), hearing loss (77.7%), and pruritus (57.4%) have been reported as the most common symptoms in patients suffering from otomycosis [1, 23, 24]. 

Our results are inconsistent with those of other studies [2, 5, 21, 25]. These differences can be due to the type of pathogenic etiological agents, anatomy of the ear, immune system status, and tympanic membrane perforation. In the current study, *A. flavus* (32.2%) was recognized as the most common cause of disease, followed by *A. niger* (29.9%) and *Candida* species (21.8%). Overall, *A. niger* and *Candida* species are reported to have a high frequency in otomycosis [5]. 

In a retrospective study performed in Mexico (2006), Arazia et al. showed that 63.9% and 26.8% of the patients with otomycosis were infected with *Aspergillus* and *Candida* *albicans*, respectively [26]. In the Middle East countries, such as India, Pakistan, Iraq, Saudi Arabia, Turkey, and Iran, *A. flavus* has been reported in invasive and noninvasive infections most frequently [27, 28]. This may be related to the weather and agricultural conditions of these countries [29]. Our results indicated that combination therapy with ceftizoxime and clotrimazole can be a good therapeutic option for otomycosis in our area due to its low cost and effectiveness.

## Conclusion

According to the results of the present study, the group receiving a combination of antifungal drugs and antibiotics demonstrated significantly better clinical outcomes than those using the anti-fungal drug alone. Therefore, the concurrent use of two drugs can be effective in the management of treatment-resistant otomycosis. 

One of the important findings of the present study was that the insufflation of ceftizoxime powder in the middle ear of the patients receiving combination therapy in the first 2-3 days of treatment resulted in the drainage of a large amount of pus, dry ear, and no discharge. This was due to the fact that this measure led to an increase in the concentration of mucus, creation of negative pressure, and discharge of the secretions contained in the aditus to mastoid antrum, which in turn results in faster dry ear.
